# Influence of Catecholamines (Epinephrine/Norepinephrine) on Biofilm Formation and Adhesion in Pathogenic and Probiotic Strains of *Enterococcus faecalis*

**DOI:** 10.3389/fmicb.2020.01501

**Published:** 2020-07-24

**Authors:** Mélyssa Cambronel, Flore Nilly, Ouiza Mesguida, Amine Mohamed Boukerb, Pierre-Jean Racine, Olfa Baccouri, Valérie Borrel, Jérome Martel, Florian Fécamp, Rikki Knowlton, Kurt Zimmermann, Eugen Domann, Sophie Rodrigues, Marc Feuilloley, Nathalie Connil

**Affiliations:** ^1^Laboratoire de Microbiologie Signaux et Microenvironnement EA 4312, Université de Rouen, Normandie Université, Évreux, France; ^2^Laboratory of Protein Engineering and Bioactive Molecules, National Institute of Applied Sciences and Technology, University of Carthage, Tunis, Tunisia; ^3^SymbioPharm GmbH, Herborn, Germany; ^4^Institute of Medical Microbiology, German Centre for Infection Research, Justus-Liebig-University Giessen, Giessen, Germany

**Keywords:** *E. faecalis*, biofilm, adhesion, adrenergic sensor, VicK

## Abstract

*Enterococcus faecalis* has controversial status due to its emerging role in nosocomial infections, while some strains with beneficial effects are used as probiotics and starter cultures in dairy industry. These bacteria can be found as resident or transient germs in the gut or on skin, where they are continually exposed to various eukaryotic molecules. In this context, the aim of our work was to evaluate the effect of the catecholamine stress hormones, epinephrine (Epi), and norepinephrine (NE) on some *Enterococcus s*trains. Four *E. faecalis* strains were included in this study: *E. faecalis* MMH594 and *E. faecalis* V583, pathogenic strains of clinical origin, *E. faecalis* Symbioflor 1 clone DSM 16431, a pharmaceutical probiotic, and *E. faecalis* OB15, a probiotic strain previously isolated from Tunisian rigouta (Baccouri et al., 2019). Epi was found to modulate the formation of biofilm (biovolume and thickness) in *E. faecalis*, whether pathogens or probiotics. NE had less effect on biofilm formation of these bacteria. We also investigated the effect of Epi and NE on adhesion of *E. faecalis* to eukaryotic cells as it is the first step of colonization of the host. Epi was found to significantly enhance the adhesion of MMH594 and OB15 to Caco-2/TC7 intestinal cells and HaCaT keratinocyte cells, whereas NE significantly increased the adhesion of V583 and Symbioflor 1 DSM 16431 to Caco-2/TC7 cells, the adhesion of MMH594, Symbioflor 1 DSM 16431, and OB15 to HaCaT cells. Analysis of a putative adrenergic sensor of Epi/NE in *E. faecalis*, compared to QseC, the *Escherichia coli* adrenergic receptor, allowed the identification of VicK as the nearest protein to QseC with 29% identity and 46% similarity values. Structure modeling and molecular docking of VicK corroborated the hypothesis of possible interactions of this putative adrenergic sensor with Epi and NE, with binding energies of −4.08 and −4.49 kcal/mol, respectively. In conclusion, this study showed for the first time that stress hormones could increase biofilm formation and adhesion to eukaryotic cells in *E. faecalis*. Future experiments will aim to confirm by *in vivo* studies the role of VicK as adrenergic sensor in *E. faecalis* probiotic and pathogen strains. This may help to develop new strategies of antagonism/competition in the gut or skin ecological niches, and to prevent the colonization by opportunistic pathogens.

## Introduction

*Enterococcus faecalis*, is a Gram-positive bacterium which belongs to the Firmicutes group, one of the two most represented phyla in the gut, along with Bacteroidetes (Arumugam et al., 2011), both gathering around 90% of the 1000 species found in the intestinal microbiota (Sekirov et al., 2010). This bacterium is a normal resident of the gastrointestinal tract of many hosts as insects, birds, reptiles, and mammals (Dubin and Pamer, 2018) but can also be present in the skin (Shami et al., 2019).

Strains of *E. faecalis* have been used for a very long time in the food industry, particularly as lactic ferments in the manufacture of cheese (Giraffa et al., 1994) because of their ability to develop under conditions of high salinity and acidic pH (Wessels et al., 1990). Their proteolytic and lipolytic activities contribute to the maturing of cheeses and the production of flavors (Thompson and Marth, 1986). This germ can be found as well in other fermented foods such as sausages, olives or plants. Some strains have been proposed for food biopreservation because they produce bacteriocins (enterocins), which can inhibit the growth of pathogens in food, or the development of spoilage bacteria (Baccouri et al., 2019).

Some strains of *E. faecalis* have been used also as probiotics for humans and/or animals. Indeed, this is the case of *E. faecalis* Symbioflor 1 clone DSM 16431. This strain was first isolated from the stool of a healthy adult human in the 1950s, and then used for more than 50 years without any report of infections (Fritzenwanker et al., 2013) and now included in pharmaceutical preparations (SymbioPharm, Herborn, Germany). This probiotic formulation has been recommended for respiratory tract infections such as influenza, colds, sinusitis, otitis, and/or bronchitis. In animals, enterococcal probiotics are mainly used to treat or prevent diarrhoea, for immune stimulation or to improve growth (Franz et al., 1999).

However, some strains of *E. faecalis* can have many virulence factors and have been implicated as opportunistic pathogens in nosocomial infections, particularly endocarditis, urinary tract infections or bacteremia. The resistance of *E. faecalis* to many antibiotics and its ability to form biofilms responsible for chronic infections is currently a serious problem in hospitals, as it has been estimated that approximately 65% of hospital infections involve bacterial biofilm (Potera, 1999; Bjarnsholt, 2013). Thus, due to its virulence factors, its role as an opportunistic pathogen, and its ability to transfer antibiotic resistance genes to other microorganisms, *E. faecalis* does not have GRAS (Generally Recognized As Safe) status and its use in food requires a case-by-case study (Ogier and Serror, 2008).

Inside the human body, eukaryotic and prokaryotic cells can communicate through signal molecules, secreted by host cells. It has been well described that molecules released under stress can impact the physiological behavior of bacteria (Freestone et al., 2008). Stress or physical effort leads to the presence of catecholamines: epinephrine (Epi), norepinephrine (NE), and dopamine in the human body. These molecules have been found to improve the growth of many Gram-negative bacteria, including several pathogens like *Escherichia coli*, *Vibrio cholera*e, or *Salmonella enterica* (Lyte and Ernst, 1991; Halang et al., 2015). Epi and NE can also modulate the virulence and the biofilm formation capacity of various bacteria (Freestone et al., 2012; Hegde et al., 2009; Yang et al., 2014). However, very few studies regarding the effect of catecholamines on Gram-positive bacteria have been conducted. Sandrini et al. (2014) showed that NE was able to increase the growth and biofilm production of *Staphylococcus pneumoniae*, and the expression of genes involved in host colonization. This molecule was also found to enhance growth and biofilm in *Staphylococcus epidermidis* (Freestone et al., 1999; Lyte et al., 2003). Roberts et al. (2003) showed that growth of *Actinomyces naeslundii* and *Actinomyces gerenscseriae*, bacteria involved in periodontal diseases, was increased in presence of both NE and Epi. More recently, it has been additionally demonstrated that catecholamines (Epi and NE) had no impact on the growth of the Gram-positive bacterium *Cutibacterium acnes*, but these molecules were able to promote its biofilm formation (Borrel et al., 2019).

The sensitivity of prokaryotes to host signal molecules requires the presence of bacterial sensors (Lesouhaitier et al., 2009). For this, bacteria have developed various mechanisms, including the presence of two-component systems. In the pathogen *E. coli* O157:H7, the two-component systems QseBC and QseEF have been shown to be involved in the response to Epi and NE (Zhu et al., 2006; Rasko et al., 2008). Adrenergic sensors exist also in *S. enterica* serovar Typhimurium (Karavolos et al., 2008; Moreira and Sperandio, 2012).

Till now, to our knowledge, no sensors of Epi/NE have been identified in *E. faecalis* (or other Gram-positive bacteria). However, on skin or in gut, pathogenic, commensal or probiotic strains of *E. faecalis* can be in contact with these catecholamines at the production and/or action sites. In this context, the aim of our study was to investigate for the first time the impact of these molecules on *E. faecalis* biofilm formation and adhesion.

## Materials and Methods

### Bacterial Strains and Growth Conditions

Four *E. faecalis* strains were used in this study, two pathogenic strains: MMH594 (Huycke et al., 1991) and V583 (Sahm et al., 1989), isolated from blood, and two probiotic strains: OB15 isolated from Rigouta, a Tunisian traditional fermented dairy product (Baccouri et al., 2019) and Symbioflor 1 DSM 16431 isolated from a stool specimen of an healthy human adult (Symbiopharm, Herborn, Germany). Bacteria were routinely grown at 37°C under static conditions in brain heart infusion (BHI).

### Comparative Phylogenomic Analysis

*E. faecalis* OB15 has been obtained previously (Baccouri et al., 2019) and the genome sequences of the four strains were compared to each other to provide a robust measurement of their genetic distance. For this, the average nucleotide identity (ANI) and the genome-to-genome distance calculator (GGDC) were analyzed using PYANI v0.2.7, and isDDH, respectively^1^.

### Biofilms on Polystyrene Surface

*E. faecalis* strains were grown in 96-wells microplates under static conditions, in BHI medium, with or without Epi or NE (1 or 100 μM) for 24 h at 37°C. At the end of incubation, microplates were washed three times with water, then biofilms were stained with 0.1% crystal violet for 15 min and dried 10 min at 37°C. Crystal violet was then resuspended with 100 % ethanol and OD was measured at 595 nm using a multiple plate reader Spark (Tecan).

### Biofilms on Glass Surface

*E. faecalis* strains were grown in 24-well glass-bottom microplates under static conditions, in BHI medium containing 1% glucose, with or without Epi or NE (1 μM), for 24 h at 37°C. At the end of incubation, microplates were washed three times with sterile physiological water, stained with 5 μM of SYTO^®^ 9 green fluorescent nucleic acid stain (Invitrogen, Thermo Fisher Scientific) for 15 min, then washed twice and observed by confocal laser scanning microscopy CLSM.

### Confocal Laser Scanning Microscopy

Biofilm observations were performed with a Zeiss LSM710 (Zeiss, Germany) using a 40× oil immersion objective. Bacteria were detected by monitoring the SYTO^®^ 9 green fluorescence. This fluorochrome was excited at 488 nm and fluorescence emission was detected between 500 and 550 nm. Images were taken every micrometer throughout the whole biofilm depth. For visualization and processing of three-dimensional (3D) image data, the Zen 2.1 software (Zeiss, Germany) was used. Quantitative analyses of image stacks were performed using the COMSTAT 2 software^2^ (Heydorn et al., 2000; Vorregaard, 2008). At least three image stacks from each of three independent experiments were used for each analysis.

### Cell Culture

Caco-2/TC7 colon adenocarcinoma cells and HaCaT keratinocyte cells were grown in DMEM (Dulbecco’s Modified Eagle Medium) containing 15 and 10% of heat-inactivated fetal bovine serum (FBS) respectively, and penicillin/streptomycin (100 μg/mL). Cells were cultivated at 37°C, in 5% CO_2_ – 95% air atmosphere, and the medium was regularly changed. For adhesion assay, cells were seeded in 24-well culture plates treated for tissue culture and were used at confluence.

### Adhesion Assay

*E. faecalis* strains were grown for 2 h with or without 1 μM Epi or NE, and then bacterial cells were centrifuged at 8000 × *g* for 10 min. The cell pellets were resuspended in DMEM without FBS and antibiotics, at a concentration of 10^8^ CFU/mL, and applied on the confluent cell monolayers. After 2 h of incubation, Caco-2/TC7 or HaCaT cells were washed twice with PBS (Phosphate Buffer Saline) to remove non adherent bacteria, and then disrupted with 0.1% Triton 100×. The lysates were then diluted and plated on BHI agar, to determine the number of adherent bacteria.

### *In silico* Sequence Analysis of Putative Adrenergic Sensors

Sensors homolog to QseC, the bacterial adrenergic sensor identified in *E. coli* strain K-12 (P40719.2), were screened in our *E. faecalis* genome sequences using BLASTp with default parameters. CLUSTALX2 v.2.1 was used for multiple alignment of the obtained protein sequences. The created aligned file was visualized with Jalview 2 (Waterhouse et al., 2009).

### Structure Prediction and Modeling

Physical and chemical characteristics of *E. faecalis* VicK (WalK) putative adrenergic sensor were analyzed by ExPASy-ProtParam tool^3^ (Gasteiger et al., 2005). Conserved domains in this protein were detected using the Conserved Domains Search tool^4^ (Lu et al., 2020). The online server PSIPred^5^, was used to predict the 2D-structure and the subcellular localization of VicK (WalK) (McGuffin et al., 2000).

The 3D-structure of VicK (WalK) was predicted by using Raptor X^6^ (Källberg et al., 2012) and Modeller (Sali and Blundell, 1993). The models generated by Modeller were submitted to the energy function DOPE (Shen and Sali, 2006). PyMOL was used to validate a model, among others and PDBsum^7^ allowed us to have an overview of the content of each 3D structure (Laskowski et al., 2018).

### Molecular Docking

Molecular docking of the putative sensor VicK (WalK) with various ligands: Epi, NE, phentolamine, a reversible, nonselective α-adrenoceptor antagonist (Clarke et al., 2006; Laverty et al., 2014), and propranolol, a β-adrenoceptor antagonist (Zhou et al., 2011), was performed by AutoDock 4.2 (Morris et al., 2009) that has been found as the most cited docking program in previous studies (Sousa et al., 2006; Chen, 2015). The structures of Epi (CID: 5816), NE (CID: 439260), phentolamine (CDI: 5775), and propranolol (CID: 4946) were downloaded from the PubChem database^8^ in SDF format. The specified 3D structure of the ligands in SDF format were converted into PDB format using PyMOL^9^.

All the detailed steps of structure prediction, modeling, and molecular docking of VicK (WalK) are presented in a supplementary data organigram (Supplementary Figure S1).

### Statistical Analysis

Data are expressed as means ± standard error (SE) of minimum three experiments done in triplicate (*n* = 9). GraphPad Prism 8.1.2 and paired *t*-test were used to compare data between treated and untreated groups, and the means within the same set of experiments. Statistical significance was determined at *P* < 0.05.

## Results

### Average Nucleotide Identity Analysis

**FIGURE 1:**
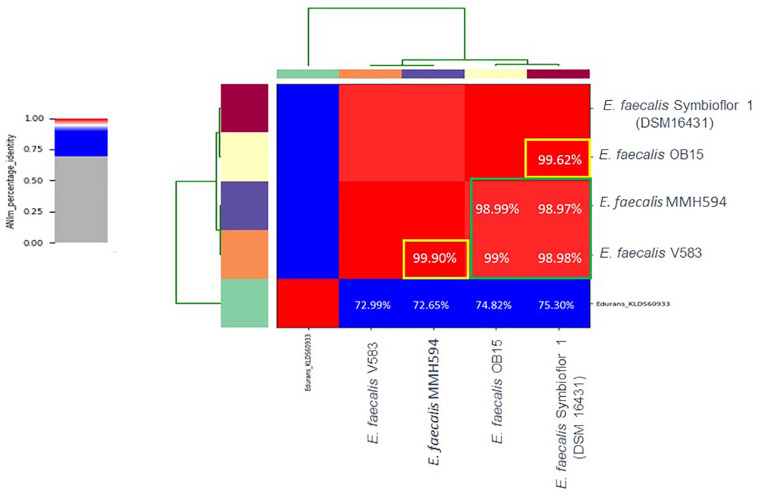
Heat-map of Average Nucleotide Identity (ANI) between *E. faecalis* Symbioflor 1 clone DSM 16431, *E. faecalis* OB15, *E. faecalis* MMH594, and *E. faecalis* V583.

Nucleotide-level genomic similarity between coding-regions of the four genomes, with *Enterococcus durans* strain KLDS6093 (CP012384.1) as an outliner, showed that the two probiotics *E. faecalis* OB15 and *E. faecalis* Symbioflor 1 DSM 16431 (NC 019770.1) were close to each other (Figure 1) in terms of genomic distance (99.62%), and also the two pathogens MMH594 and V583 (99.90%). Conversely, the distance between the probiotics and the pathogens strains was greater (98.97–99%).

### Biofilm Quantification by Crystal Violet

**FIGURE 2:**
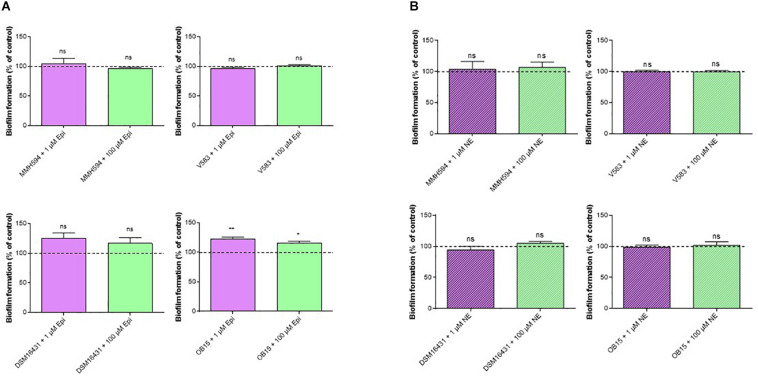
Effect of Epi **(A)** and NE **(B)** on biofilm formation of *E. faecalis* on polystyrene surface, quantified by crystal violet. *n* = 12. ns, not significant; *P < 0.05, **P < 0.01.

A polystyrene surface was used to study the impact of Epi and NE on *E. faecalis* biofilm production on abiotic surface (Figure 2). The results showed that biofilm formation of the pathogenic strains (MMH594 and V583) were not modified by a treatment with 1 or 100 μM Epi (Figure 2A). On the contrary, this substance slightly enhanced the biofilm formation of the probiotic strains. The modulation observed for *E. faecalis* Symbioflor 1 DSM 16431 was not statistically significant, but for OB15 the biofilm increased by 22 ± 2% when the bacterium was treated with 1 μM Epi, and by 15 ± 1% for 100 μM. Figure 2B shows the results obtained with NE. This catecholamine was found to have no effect in our experimental conditions on the biofilm formation on polystyrene surface of the four strains of *E. faecalis* studied.

### Biofilm Analysis and Quantification by Confocal Laser Scanning Microscopy

**FIGURE 3:**
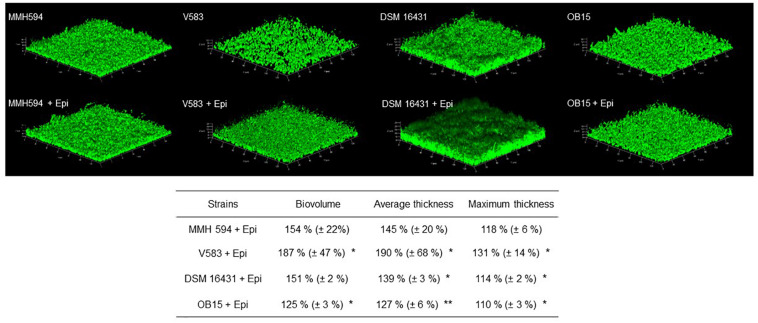
Effect of Epi on biofilm formation (biovolume, average thickness, and maximum thickness) of *E. faecalis* on glass surface, measured by SYTO^®^ 9 green fluorescent nucleic acid staining and confocal laser scanning microscopy (CLSM). *n* = 9. *P < 0.05, **P < 0.01.

**FIGURE 4:**
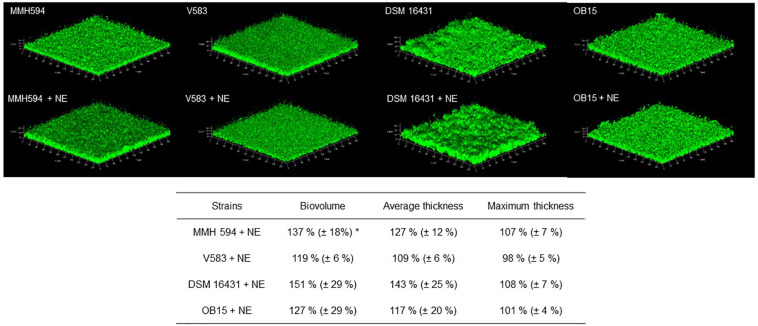
Effect of NE on biofilm formation (biovolume, average thickness, and maximum thickness) of *E. faecalis* on glass surface, measured by SYTO^®^ 9 green fluorescent nucleic acid staining and confocal laser scanning microscopy (CLSM). *n* = 9. *P < 0.05.

As quantification of biofilms with crystal violet is not a precise test, biofilm formation of *E. faecalis* strains, treated or not with 1 μM of Epi or NE were then studied on glass surface and measured by CLSM (Figures 3, 4). The results showed that 3D structure of *E. faecalis* biofilms was homogeneous except for Symbioflor 1 DSM 16431 which was not, and quite different from the three other strains studied, as can be seen in Figures 3, 4. Three biofilm parameters were estimated (biovolume, average and maximum thicknesses). The results showed that Epi (Figure 3) significantly increased the biovolumes of the pathogenic strain V583 (+87 ± 47%) and the probiotic strain OB15 (+25 ± 3%), the average and maximum thicknesses of V583 (+90 ± 68%, +31 ± 14%), and of the two probiotic strains Symbioflor 1 DSM 16431 (+39 ± 3%, +14 ± 2%) and OB15 (+27 ± 6%, +10 ± 3%), compared to the biofilm values obtained with the untreated control bacteria. An increase of these parameters was also observed for *E. faecalis* MMH594 biofilms after treatment with Epi, but the values obtained were not statistically significant compared to control without the molecule. The results of the same experiment conducted with NE are visible in Figure 4. This catecholamine also led to a small increase of biofilms (biovolume, average and maximum thicknesses) of the strains tested.

### Adhesion on Eukaryotic Cells

**FIGURE 5:**
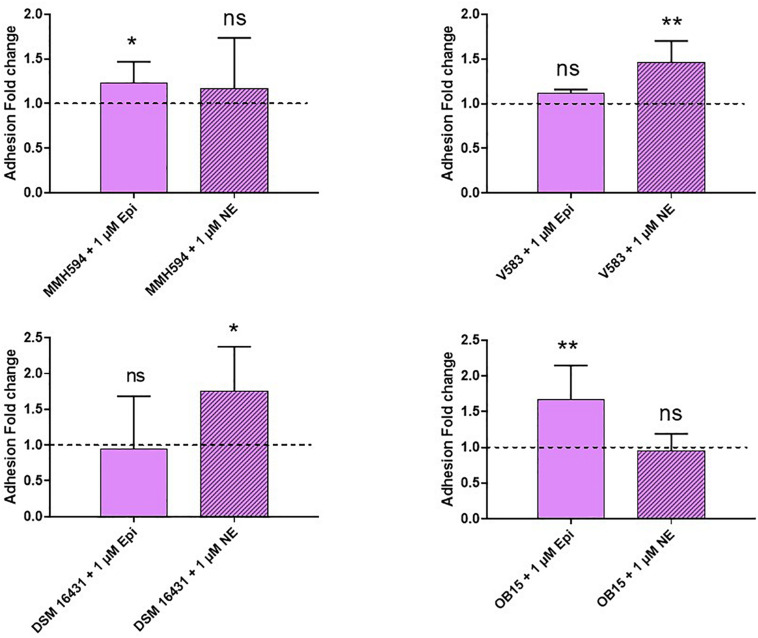
Effect of Epi and NE on adhesion of *E. faecalis* (MMH594, V583, DSM 16431, and OB15) to Caco-2/TC7 intestinal cells. *n* = 9. ns, not significant; *P < 0.05, **P < 0.01.

**FIGURE 6:**
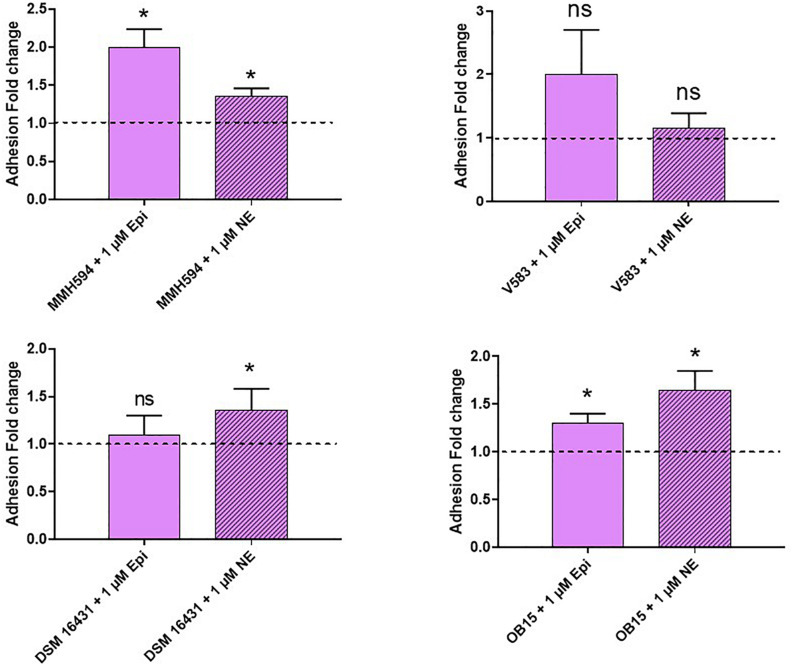
Effect of Epi and NE on adhesion of *E. faecalis* (MMH594, V583, DSM 16431, and OB15) to HaCaT keratinocyte cells. *n* = 9. ns, not significant; *P < 0.05.

Pathogenic and probiotic strains of *E. faecalis* were treated for 2 h with 1 μM Epi or NE, then incubated on Caco-2/TC7 intestinal cells to study bacterial adhesion. The results obtained are presented on Figure 5. Epi was found to enhance significantly the adhesion of *E. faecalis* MMH594 (fold change of 1.23) and *E. faecalis* OB15 (fold change of 1.66). NE increased the adhesion of *E. faecalis* V583 (fold change of 1.46) and *E. faecalis* Symbioflor 1 DSM 16431 (fold change 1.75). The same experiment has been conducted using HaCaT keratinocyte cells as model (Figure 6). As previously found for Caco-2/TC7 cells, a treatment of the bacteria with 1 μM Epi led to a significant enhancement of adhesion for *E. faecalis* MMH594 (fold change of 2) and *E. faecalis* OB15 (fold change of 1.45). An increase was also observed for *E. faecalis* V583, but this variation was not statistically significant due to disparate values. NE increased the adhesion of *E. faecalis* MMH594 (fold change of 1.46), *E. faecalis* Symbioflor 1 DSM 16431 (fold change of 1.40) and *E. faecalis* OB15 (fold change of 1.52).

### *In silico* Analysis of Putative Adrenergic Sensors

**FIGURE 7:**
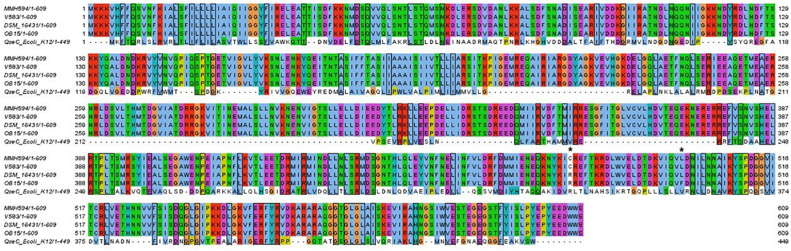
Multiple alignment of the four VicK amino acid sequence with QseC from *E. coli* strain K-12. Shared amino acids are boxed. Asterisks indicated different amino acids between *E. faecalis* strains.

Sensors homolog to QseC, the *E. coli* adrenergic sensor, were investigated in the four *E. faecalis* strains of the study (Figure 7). BLASTp analysis allowed the identification of VicK (WalK) as the nearest protein to QseC with 29% identity and 46% similarity values. Multiple alignment showed that VicK sequence is well conserved within the four *E. faecalis* strains studied, with identification of variants. Indeed, we found two amino-acid changes at positions 478 (Cys for pathogens, and Arg for probiotics and QseC at position 336), and 499 (Leu for the two pathogens and DSM 16431, Phe for OB15 and Val for QseC at position 357).

As the sequence of VicK (WalK) showed high identity among the four strains of *E. faecalis* studied, we decided to focus on OB15 sequence for the next steps of modeling and docking.

### Structure Modeling

#### Physicochemical Properties

The physicochemical proprieties of amino acid sequence are important to know the structural and functional proprieties of a protein (Gasteiger et al., 2005). Using ProtParam, we found for *E. faecalis* OB15 putative adrenergic sensor VicK (WalK), a computed molecular weight of 69661.02 kDa, a theoretical isoelectric point (pI) of 4.97, 99 amino acids negatively charged (Asp+Glu) and 74 amino acids positively charged (Arg+Lys). The instability index (II) was computed to be 30.93, which allows to classify VicK (WalK) as a stable protein.

#### Conserved Domains

Conserved domains in *E. faecalis* VicK (WalK) were detected by NCBI Conserved Domains Search. The results showed a conserved domain homologous with the cell wall metabolism sensor histidine kinase WalK found in *Staphylococcus aureus*, which is part of a two-component system with partner protein WalR. Thus, *E. faecalis* VicK (WalK) adrenergic putative sensor can be classified in the superfamily of HKWalK.

#### 2D and 3D Modeling of VicK (WalK)

**FIGURE 8:**
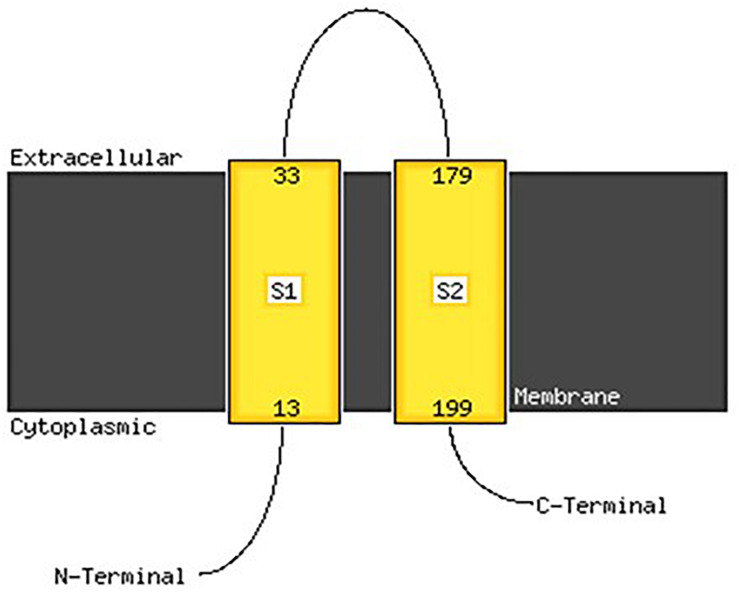
Membrane localization of VicK (WalK) of *E. faecalis* OB15 using the PSIPred server. An extracellular motif is revealed along with 146 amino acids, between the amino acid 33 and 179, that may represent the domain that can bind catecholamines.

Analysis with PSIPred program (McGuffin et al., 2000) showed that VicK (WalK) consists of 48.60% α-helix, 33.99% β-sheets and 17.41% random coil. MEMSAT-SVM program detected two transmembrane regions between amino acids 13 and 33 and between amino acids 179 and 199. The carboxy-terminus and the amino terminus were predicted to be in the cytoplasm while the sequence between the amino acid 33 and 179 is an extracellular domain, thus representing the region of VicK (WalK) that may be able to interact with the catecholamines ligands, and will be called VicK_ex_ (WalK_ex_) in the rest of this manuscript (Figure 8).

The 3D-structure of the extracellular domain VicK_ex_ (WalK_ex_) of the VicK (WalK) putative adrenergic sensor of *E. faecalis* OB15 was then predicted by the homology modeling servers Raptor X. and Modeller, using WalK_ex_ of *S. aureus* as template. Several models were obtained by these programs and evaluated to select the best predictive model to be used for molecular docking.

#### Molecular Docking

**FIGURE 9:**
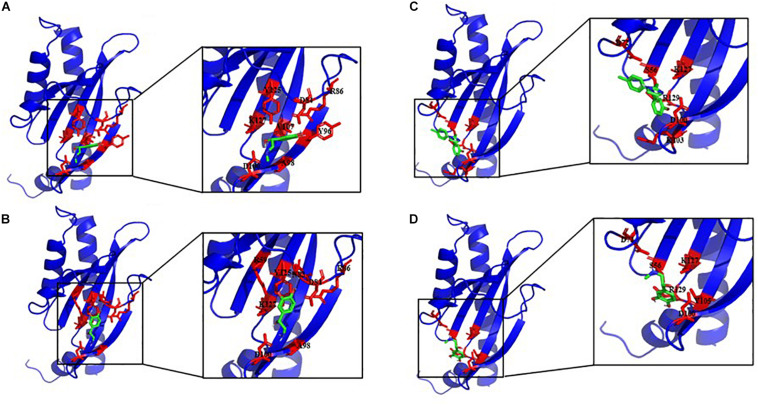
Best docking positions of Epi **(A)**, NE **(B)**, phentolamine **(C)**, and propranolol **(D)** to the putative structure of VicK_ex_ (WalK_ex_) of *E. faecalis* OB15. Binding site residues are shown in red.

To study if *E. faecalis* VicK (WalK) putative adrenergic sensor would be able to bind Epi, NE, and the adrenergic blockers phentolamine and propanolol, a molecular docking analysis was performed using AutoDock 4.2. and the predictive model generated before for VicK_ex_ (WalK_ex_). A total of 50 conformations were obtained for each ligand, with a different number of clusters. Because AutoDock considers the enthalpic binding energy in its search for favorable orientations of torsionally flexible ligands, the intermolecular interaction energy function is not necessarily accurate in predicting the true binding energy. Thus, the cluster with the absolute minimum energy does not automatically represent the best or global minimum energy of the ligand in the receptor binding-site (Nyrönen et al., 2001). Consequently, taking into account the number of possible conformations for each cluster, the binding energy and the residues involved in the interaction site, a cluster among others was selected to be the best putative interactions site of *E. faecalis* VicK_ex_ (WalK_ex_) putative adrenergic sensor with the different ligands. Table 1 and Figure 9 show that Asp100 (D100) and Lys127 (K127) amino acids are both involved in the interaction site for the four ligands tested. Six out of eight residues implicated in the interaction site are the same for Epi and NE: Asp84 (D84), Arg86 (R86), Ala98 (A98), Asp100 (D100), Tyr125 (Y125), and Lys127 (K127). Phentolamine and propanolol both interact in a cluster which involves Ser56 (S56), Asp72 (D72), Asp100 (D100), Lys127 (K127), and Asn129 (R129). Only one amino acid differs in the interaction cluster between phentolamine and propanolol with Lys103 (K103) for phentolamine and Val105 (V105) for propanolol. Docking analysis also showed that three hydrogen bonds are involved in the interaction between *E. faecalis* VicK_ex_ (WalK_ex_) and Epi/NE. Two residues in this hydrogen bonds are common for these ligands, Asp84 and Asp100, whereas the third bond involves an Arg amino acid, at a different position, Arg86 for Epi and Arg59 for NE.

**TABLE 1:** The binding energy, number of conformations in the selected cluster and the amino acids involved in the interaction site for the four ligands tested.

	Binding energy kcal/mol	Number of conformations	Amino acids involved in the interaction site
Epinephrine	−4.08	2	Asp84; Arg86; Ala98; Tyr96; Asp100; Val107; Tyr125; Lys127
Norepinephrine	−4.49	4	Arg59; Asn83; Asp84; Arg86; Ala98; Asp100; Tyr125; Lys127
Phentolamine	−3.5	2	Ser56; Asp72; Asp100; Lys103; Lys127; Asn129
Propanolol	−4.39	5	Ser56; Asp72; Asp100; Val105; Lys127; Asn129

## Discussion

Bi-directional dialogue between host and microbe have now been studied for more than two decades and this has led to the proposal of the field of microbial endocrinology (Lyte, 1993). Many eukaryotic molecules (neurotransmitters, immune modulators, and peptide hormones) can be sensed by bacteria in the host (Lesouhaitier et al., 2009), and among these substances the effect of catecholamines (Epi/NE) has been widely investigated. These stress hormones have been shown to be able to stimulate the growth of some Gram-negative pathogens such as *E. coli*, *V. cholera*, *Helicobacter pylori*, *S. enterica* and *Yersinia enterocolitica*, hence promoting virulence and the establishment of infection (Sarkodie et al., 2019). Since catechol rings are known siderophores in bacteria, catecholamines have been shown to serve as auxiliary siderophores that enhance growth of some Gram-negative bacteria by improving iron uptake in growth-limiting media (Kinney et al., 2000). Stress hormones, Epi and NE, can also affect the growth of anaerobic bacteria such as *Fusobacterium nucleatum*, *Prevotella* spp., *Porhyromonas* spp., *Tanerella forsythia*, and *Propionibacterium acnes* (Boyanova, 2017). The effects of Epi and NE are species-specific, sometimes strain-specific and can lead to a growth increase or decrease, or no effect on the growth (Belay et al., 2003). Till now, very few data are available on Gram-positive bacteria, so we decided to investigate the effect of Epi and NE on *E. faecalis* as this germ can be in contact with these molecules in gut or on the skin. For this, we selected four strains of *E. faecalis*, two pathogens: MMH594 (Huycke et al., 1991) and V583 (Sahm et al., 1989), and two probiotics: OB15 (Baccouri et al., 2019) and Symbioflor 1 DSM 16431 (Fritzenwanker et al., 2013). The genomic distances between these bacteria were first measured using ANI and the Genome-to-Genome Hybridization similarity methods. As could be expected, the results obtained showed that the two probiotics *E. faecalis* OB15 and *E. faecalis* Symbioflor 1 DSM 16431 were close to each other in terms of genomic distance (99,62%), also the two pathogens MMH594 and V583 (99.90%), whereas the distance between the probiotics and pathogens strains was greater (98.97–99%). In the following experiments, the effect of Epi or NE on *E. faecalis* biofilm formation and adhesion has been investigated for these four strains, in order to see if this behavior seems to be strain specific. Quantification by crystal violet of *E. faecalis* biofilms grown on polystyrene surface showed that Epi (1 or 100 μM) only modulates biofilm formation of the probiotic strains OB15 and Symbioflor 1 DSM 16431, whereas NE had no effect in our experimental conditions whatever the strains used for this test. As quantification of biofilms by crystal violet is not a precise experiment, we then examined the structures of *E. faecalis* biofilms by CLSM after growth of the bacteria on glass surfaces with or without treatment with 1 μM of catecholamines (Epi or NE). Three parameters of the biofilms were estimated (biovolume, average and maximum thicknesses), and the results showed that Epi was able to modulate these values significantly or not, in almost all the four bacteria tested, whether pathogens or probiotics. On the contrary, NE had less effect on biofilm formation of these bacteria. In the literature, the capacity of catecholamines to enhance biofilms has been previously observed for several Gram-negative bacteria, including *E. coli* O157:H7 (Bansal et al., 2007), *Vibrio harveyi* (Yang et al., 2014) and *Pseudomonas aeruginosa* (Freestone et al., 2012; Cambronel et al., 2019). Few studies have been conducted on Gram-positive bacteria. Catecholamines have been found to enhance biofilm production in *S. pneumoniae* (Sandrini et al., 2014) *S. epidermidis* (Freestone et al., 1999; Lyte et al., 2003), and *C. acnes* (Borrel et al., 2019). Growth of *A. naeslundii* and *A. gerenscseriae*, was increased in presence of both NE and Epi (Roberts et al., 2003). On the contrary, in our study, catecholamines were found to have no effect on *E. faecalis* growth (data not shown). As the first step of biofilm formation during colonization of the host is adhesion, we then decided to investigate the effect of Epi and NE on adhesion of *E. faecalis* to eukaryotic cells. Epi was found to significantly enhance the adhesion of *E. faecalis* MMH594 and *E. faecalis* OB15 to Caco-2/TC7 intestinal cells, whereas NE only significantly increased the adhesion of *E. faecalis* V583 to these cells. Epi also promoted the adhesion of *E. faecalis* MMH594 and *E. faecalis* OB15 on HaCaT keratinocyte cells. Analysis of a putative adrenergic sensor of Epi/NE in *E. faecalis*, compared to QseC, the *E. coli* adrenergic receptor, allowed the identification of VicK as the nearest protein to QseC with 29% identity and 46% similarity values. This protein is part of the two-component system VicKR also known in the literature as WalKR or YycGF. This two-component system also exists in other Gram-positive bacteria, and has been described for example, as essential for the bacterial growth of *B. subtilis*, and bacterial wall metabolism in *S. aureus* (Dubrac et al., 2007; Dhiman et al., 2014).

Modeling of VicK (WalK) structure and molecular docking allowed us to study the interaction between this *E. faecalis* adrenergic putative sensor and Epi/NE but no data are available in the scientific literature to compare our results with known natural ligands. However, in many scientific publications, it has been demonstrated that Asp and Arg interact with NE by hydrogen bonds (Mans et al., 2007; Calvo et al., 2009; Huschmann et al., 2016). Another work conducted by Ring et al. (2013) showed that the amino acid Asp of the β2-adrenoceptor links to Epi by a hydrogen bond. In addition, the amino acids Tyr, Arg, Asn, Asp, Ala, and Lys were found to be involved in the interaction site of NE (Erlandsen et al., 1998; Huschmann et al., 2016), and the amino acids Asp, Asn, and Val, are generally found in interaction with Epi (Temperini et al., 2007; Ring et al., 2013).

Dimić et al. (2020) showed that NE binds to the β1-adrenergic receptor and β2-adrenergic receptor with a free binding energy of −6.4441 and −5.3072 kcal/mol, respectively. These results are consistent with our data, as we found that the binding energy value of the interaction between Epi and VicK_ex_ (WalK_ex_) is −4.08. This data is also in agreement with the study of Parveen et al. (2012), which have indicated values of −4.56 Kcal/mol for Epi. In the same paper, the binding energy between NE and its receptor was measured to be −8.56 Kcal/mol, a value significantly different from our result. This difference is probably explained by the fact that NE provided the best binding affinity with human Tyrosine hydroxylase (Parveen et al., 2012). For phentolamine and propanolol, the two clusters found are relatively the same, so we can suggest that these two molecules can interact with our putative adrenergic sensor VicK (WalK) and be blockers of the sensor. The values of the interaction energy of NE, Epi and propanolol are very close as we find in our study and in the literature (Parveen et al., 2012; Dimić et al., 2020). This leads to think that these three molecules may have the same affinity for VicK (WalK). This is not the case for phentolamine, the β-adrenoceptor antagonist, for which the interaction energy is higher. Thus, the affinity of this blocker toward VicK (WalK) may be lower than propranolol, the α-adrenoceptor antagonist.

## Conclusion

Taken together, the results of our study showed for the first time that catecholamines (Epi and NE) can modulate not only the biofilm formation and adhesion of pathogenic bacteria but also probiotic strains. Thus, catecholamines in human body may participate in competition for the ecological niche by acting both on pathogens and probiotics. Future works will help to confirm the role of VicK as the sensor of these molecules in *E. faecalis.*

## Data Availability Statement

The datasets generated for this study are available on request to the corresponding author.

## Author Contributions

NC designed and supervised the project. MC, FN, and SR carried out most of the experiments and analyzed the data. OB, JM, and FF participated in biofilm quantification by crystal violet. SR performed the biofilm analysis by Confocal Laser Scanning Microscopy (CLSM). VB participated in the adhesion assay on eukaryotic cells. AB conducted genome sequencing of *E. faecalis* OB15 and *E. faecalis* MMH594 and helped for bioinformatic analysis. OM and P-JR performed modeling and molecular docking. MC, AB, OM, P-JR, RK, MF, and NC wrote and revised the manuscript. All authors approved the final version of the manuscript.

## Conflict of Interest

KZ was employed by SymbioPharm GmbH and provided the *E. faecalis* Symbioflor 1 strain for the experiments. The remaining authors declare that the research was conducted in the absence of any commercial or financial relationships that could be construed as a potential conflict of interest.

## Footnotes

1 http://ggdc.dsmz.de/ggdc.php

2 http://www.imageanalysis.dk/

3 http://expasy.org/tools/protparam.html

4 http://www.ncbi.nlm.nih.gov/Structure/cdd/wrpsb.cgi

5 http://bioinf.cs.ucl.ac.uk/psipred

6 http://raptorx.uchicago.edu/StructurePrediction/

7 http://www.ebi.ac.uk/pdbsum

8 https://pubchem.ncbi.nlm.nih.gov

9 http://www.pymol.org/
