# Supplementary material for: Influence of Catecholamines (Epinephrine/Norepinephrine) on Biofilm Formation and Adhesion in Pathogenic and Probiotic Strains of Enterococcus faecalis
Source: Front Microbiol. 2020 Jul 24;11:1501. doi: 10.3389/fmicb.2020.01501 (PMC7396564; doi:10.3389/fmicb.2020.01501)
Supplement: Supplementary file 1 [file Data_Sheet_1.docx]

Best 3D model

Molecular Docking by AutoDockTools

**Fig S1**: Organigram of structure prediction, modelling and molecular docking of VicK (WalK)
